# Computer-Aided Assessment of Displacement and Reduction of Distal Radius Fractures

**DOI:** 10.3390/diagnostics11040719

**Published:** 2021-04-18

**Authors:** Yuichi Yoshii, Yasukazu Totoki, Atsuo Shigi, Kunihiro Oka, Takeshi Ogawa, Tsuyoshi Murase, Tomoo Ishii

**Affiliations:** 1 Ibaraki Medical Center, Department of Orthopaedic Surgery, Tokyo Medical University, Ami 300-0395, Japan; ishitom@tokyo-med.ac.jp; 2 Department of Orthopaedic Surgery, University of Tsukuba Hospital, Tsukuba 305-8576, Japan; yasu.am10oo@gmail.com (Y.T.); ogawat@md.tsukuba.ac.jp (T.O.); 3 Department of Orthopaedic Surgery, Osaka University, Suita 565-0871, Japan; bassman_german@yahoo.co.jp (A.S.); okakunihiro@gmail.com (K.O.); tmurase-osk@umin.ac.jp (T.M.)

**Keywords:** computer-aided diagnosis, computed tomography, distal radius fracture, osteosynthesis, three-dimensional

## Abstract

This study aims to investigate displacements and reductions of distal radius fractures using measurement indices based on the computer-aided three-dimensional (3D) radius shape model. Fifty-two distal radius fracture patients who underwent osteosynthesis were evaluated with pre- and post-operative distal radius 3D images. In the 3D images, three reference points, i.e., the radial styloid process (1), sigmoid notch volar, and dorsal edge (2) (3) were marked. The three-dimensional coordinates of each reference point and the barycentric coordinates of the plane connecting the three reference points were evaluated. The distance and direction moved, due to the reductions for each reference point, were (1) 12.1 ± 8.1 mm in the ulnar-palmar-distal direction, (2) 7.5 ± 4.1 mm in the ulnar-palmar-proximal direction, and (3) 8.2 ± 4.7 mm in the ulnar-palmar-distal direction relative to the preoperative position. The barycentric coordinate moved 8.4 ± 5.3 mm in the ulnar-palmar-distal direction compared to the preoperative position. This analyzing method will be helpful to understand the three-dimensional direction and the extent of displacements in distal radius fractures.

## 1. Introduction

A distal radius fracture is one of the most common fractures in the human body. Conventionally, plain radiographs were used for the treatment decisions. However, it has been shown that the reliability in measuring the volar tilt, radial inclination, and radial shortening on radiographs is low and that these methods are not precise enough to make treatment decisions or evaluate outcomes in distal radius fractures [1,2]. Recently, computed tomography (CT) images have been commonly used in the planning of treatment for distal radius fractures, especially, in severely displaced or comminuted fractures. CT images provide accurate evaluations of fracture types and are helpful for planning the fracture reductions [3,4,5,6,7,8,9]. Although it has been suggested that the acquisition of CT imaging data and morphometrical analysis must be standardized, there is no universal method for three-dimensional (3D) evaluations of distal radius fractures [10,11]. This makes it difficult to understand how much the fracture has been displaced and how much the fracture fragments need to move for the reductions. Even though the 3D shape of distal radius fractures has been evaluated intensively [12,13], most of the articles only discuss fracture patterns of the joint surface. There are no methods to assess the displacement and directions of the fractures quantitatively. To assess the displacement degree and reduction shape of distal radius fractures three-dimensionally, we developed a computer-based analysis method. The objective of this study is to investigate the direction and extent of displacements in distal radius fractures using measurement indices based on reference points on the 3D model and evaluate the relationship with conventional X-ray measurements.

## 2. Materials and Methods

This study protocol was approved by our Institutional Review Board. The study protocol conformed to the ethical guidelines of the 1975 Declaration of Helsinki. This was a retrospective case control study (level of evidence III). Fifty-two wrists of 52 distal radius fracture patients who underwent osteosynthesis (38 females, 14 males, mean age 63.3 years, age range 18–91) were evaluated. All patients were treated with volar locking plates. Patients were excluded if they reported a previous history of traumatic arm injuries. For the assessment of CT images, written consent was obtained from all study participants. The patients lay down with their arms elevated over their heads and their wrists and forearms in neutral positions. CT images were taken before surgery and one month after the surgery with tube settings of 120 kV and 100 mAS and a section thickness of 0.8 mm and a pixel size of 0.3 × 0.3 mm (Sensation Cardiac, SIEMENS, Malvern, PA, USA). According to preoperative X-rays (posterior-anterior and lateral view) and CT scans, fractures were classified using the AO classification system.

### 2.1. 3D Image Analysis

**Figure 1:**
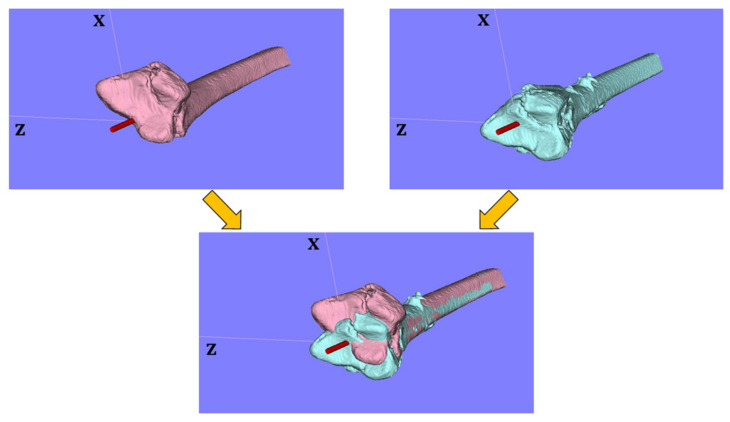
Coordinate system for the 3Dimages. The preoperative and postoperative 3D images were evaluated in the same coordinate system.

**Figure 2:**
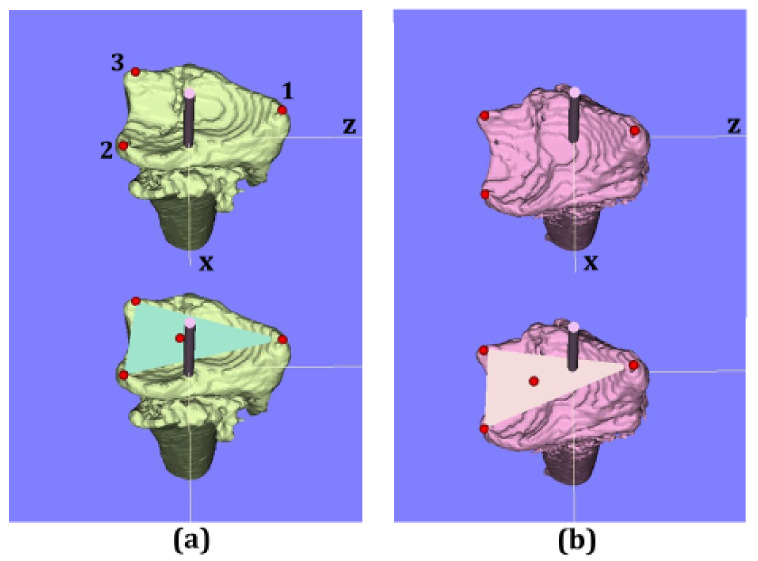
Axial view of 3D images. (**a**) Preoperative image, (**b**) postoperative image. Upper row images show three reference points. Lower row images show the barycentric coordinates of the plane connecting the three reference points. The red dots indicate radial styloid process: reference point (1), sigmoid notch volar edge: reference point (2), sigmoid notch dorsal edge: reference point (3), and the barycentric coordinates.

**Figure 3:**
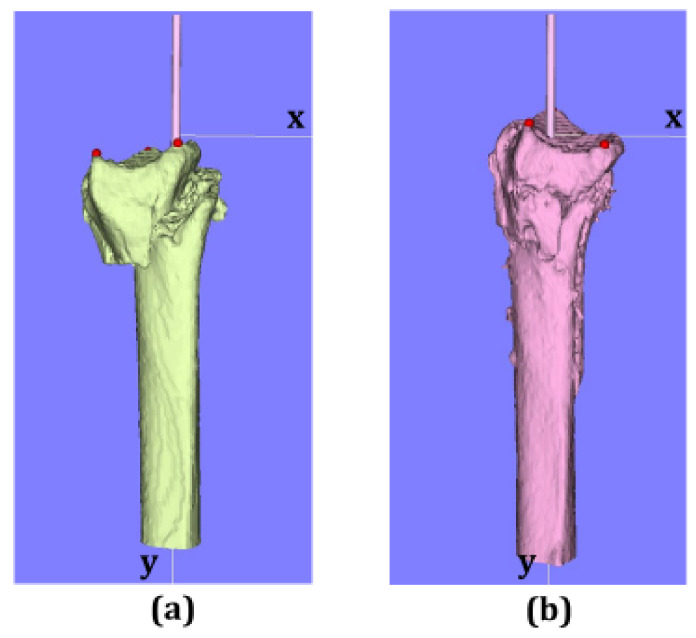
Sagittal view of 3D images. (**a**) Preoperative image, (**b**) postoperative image.

After taking the CT images, three-dimensional models of the distal radius were analyzed with image analysis software (ZedTrauma, LEXI Co., Ltd., Tokyo, Japan, and BoneSimulater, Orthree, Osaka, Japan). The Digital Imaging and Communications in Medicine (DICOM) datasets were imported into the software. The DICOM datasets were then converted into high-resolution three-dimensional image files. Each distal radius fracture was segmented semi-automatically. A three-dimensional surface model of the radius was constructed with a three-dimensional surface construction algorithm [14] in the software (Figure 1, Figure 2 and Figure 3). A coordinate system was constructed following previously recommended protocols [15,16]. The long axis of the radius was calculated from the three-dimensional surface models of the intact part of the distal radius shaft. The y-axis was defined as the long axis of the radius, and the proximal direction was defined as positive. The z-axis was parallel to the orthogonal projection of the line that initiated at the base of the distal ulnar sigmoid notch and continued to the radial styloid process on the plane perpendicular to the y-axis. The radial direction on the z-axis was defined as positive. The x-axis was normal to the yz plane. The yz plane, xy plane, and xz plane were defined as the coronal plane, sagittal plane, and axial plane, respectively. The origin of coordinates was defined with the intersection of the joint surface and radius long axis on the postoperative images. In the pre- and post-operative 3D images, three reference points were marked as follows: (1) radial styloid process, (2) sigmoid notch volar edge, and (3) sigmoid notch dorsal edge (Figure 2). The reference points (2) and (3) were defined as the points of the palmar and dorsal edges of the radius when viewed from directly above the long axis. The three-dimensional coordinates for each reference point were measured. The three-dimensional coordinates of each reference point and the barycentric coordinates of the plane connecting the three reference points were evaluated from the pre- and post-operative 3D images. For the assessment of the articular surface gap, the plane areas connecting three reference points were also measured with the pre- and post-operative 3D images.

The angle between a connecting line from the reference point (2) to the reference point (3) and a line perpendicular to the longitudinal axis of the radius was measured as the volar tilt on the 3D image (3D-VT) in the sagittal view. The angle between a line from the reference point (1) to the reference point (2) and a line perpendicular to the longitudinal axis of the radius was measured as the radial inclination on the 3D image (3D-RI) in the coronal view. Before proceeding to the further analysis, two raters (A: hand surgery specialist, B: orthopedic resident) independently measured the three reference points, and the reliability of the 3D-VT and 3D-RI measurements were evaluated for the initial 20 cases.

### 2.2. X-ray Image Analysis

X-ray images were taken for all patients at the same time with CT scanning before and after surgery. The postero-anterior view was obtained with the elbow flexed 90 degrees (the ulna perpendicular to the humerus) and the forearm in the pronated position. The lateral view was obtained with the elbow flexed 90 degrees and adducted against the trunk. The wrists were in a neutral position with no flexion, extension, or deviation in either view. Using the obtained X-ray images, the indices of radial inclination and volar tilt were measured with image analysis software (Synapse Vincent, Fujifilm Holdings Co., Tokyo, Japan). The angle between a line from the dorsal edge to the volar edge of the radius and a line perpendicular to the longitudinal axis of the radius was measured as the volar tilt (2D-VT). The angle between a line from the radial styloid tip to the ulnar aspect of the distal radius and a line perpendicular to the longitudinal axis of the radius was measured as the radial inclination (2D-RI).

### 2.3. Statistical Analysis

To assess the reliability of the measurements, the intra-class correlation coefficients (ICC) for the parameters between rater A and B measurements were evaluated. The average positions for three reference points relative to the origin were analyzed pre- and post-surgery. The results are expressed as the mean ± standard deviation. For the normality test of datasets, the Shapiro-Wilk test was used. Since the distributions of datasets were different between pre- and post-surgery, and among the reference points, non-parametric tests were used for the analysis. The movement distance with reductions for each reference point were compared using Friedman’s test followed by the Scheffe post-hoc test. The plane area connecting three reference points were compared between the pre- and post-surgery using the Wilcoxon signed-rank test. In addition, the correlations between 3D-VT and 2D-VT, and between 3D-RI and 2D-RI were analyzed with Pearson’s correlation coefficients. *p* values of <0.05 were considered significant.

## 3. Results

**Figure 4:**
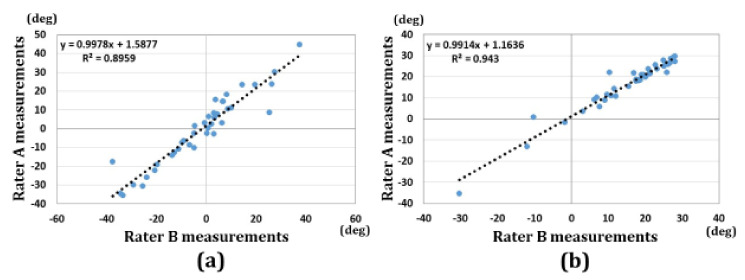
Results of correlations for the two rater measurements. (**a**) 3D-VT, (**b**) 3D-RI.

Figure 4 shows the reliability of the measurements. The ICCs for the 3D-RI and 3D-VT were 0.97 and 0.96, respectively. There were excellent correlations between the two raters’ measurements.

**Figure 5:**
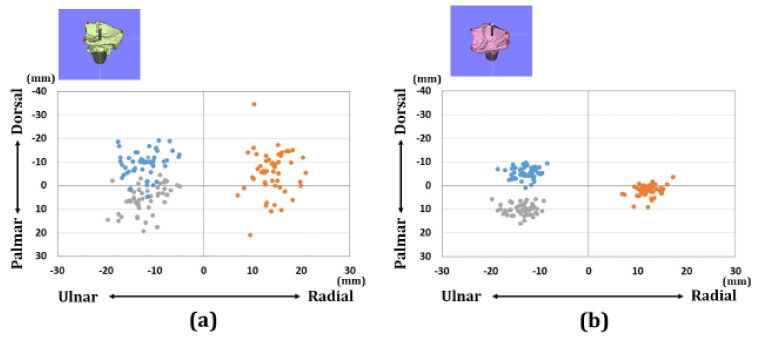
Results of coordinates for the three reference points in the axial plane. (**a**) Results of coordinates for the preoperative image. (**b**) Results of coordinates for the postoperative image. The orange dots indicate the radial styloid process: reference point (1). The gray dots indicate the sigmoid notch volar edge: reference point (2). The blue dots indicate the sigmoid notch dorsal edge: reference point (3).

**Figure 6:**
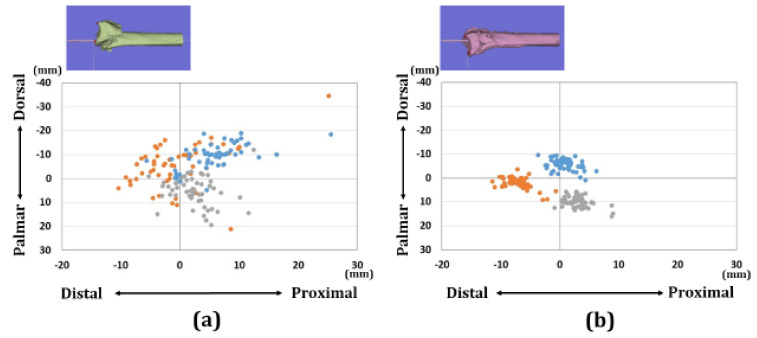
Results of coordinates for the three reference points in the sagittal plane. (**a**) Results of coordinates for the preoperative image. (**b**) Results of coordinates for the postoperative image. The orange dots indicate the radial styloid process: reference point (1). The gray dots indicate the sigmoid notch volar edge: reference point (2). The blue dots indicate the sigmoid notch dorsal edge: reference point (3).

**Figure 7:**
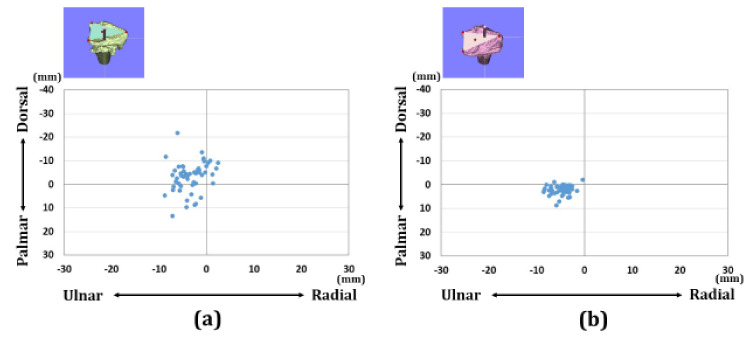
Results of barycentric coordinates of the three reference points in the axial plane. (**a**) Results of barycentric coordinates for the preoperative image. (**b**) Results of barycentric coordinates for the postoperative image.

Regarding the overall assessments, there were 13 patients with A3 fractures, 22 patients with C2 fractures, and 17 patients with C3 fractures in each group. The distributions of each reference point in the axial and sagittal planes are shown in Figure 5, Figure 6 and Figure 7. Before surgery, each reference point was located at the (1) 18.1 ± 5.1 mm radial-dorsal-distal position, (2) the 14.5 ± 4.9 mm ulnar-palmar-proximal position, (3) the 17.5 ± 4.4 mm ulnar-dorsal-proximal position to the origin. The barycentric coordinate was located at the 8.6 ± 4.3 mm ulnar-dorsal-proximal position to the origin. Thirty-nine cases were displaced in the dorsal direction. Thirteen cases were displaced in the palmar direction. After surgery, each reference point was located at the (1) 14.5 ± 1.8 mm radial-palmar-distal position, the (2) 17.3 ± 2.6 mm ulnar-palmar-proximal position, and the (3) 14.2 ± 2.1 mm ulnar-dorsal-proximal position to the origin. The barycentric coordinate was located at the 5.9 ± 1.9 mm ulnar-palmar-distal position to the origin. After surgery, each reference point moved (1) 12.1 ± 8.1 mm in the ulnar-palmar-distal direction, (2) 7.5 ± 4.1 mm in the ulnar-palmar-proximal direction, and (3) 8.2 ± 4.7 mm in the ulnar-palmar-distal direction relative to the preoperative position. The barycentric coordinate moved 8.4 ± 5.3 mm to the ulnar-palmar-distal position compared to the preoperative position. The movement distance with reductions was significantly larger in the radial styloid process compared to the sigmoid notch volar and dorsal edges (*p* < 0.01).

The plane areas connecting the three reference points were 216.9 ± 36.7 mm^2^ and 210.2 ± 35.9 mm^2^ for pre- and post-surgery, respectively. There was a significantly smaller plane area on the post-operative images compared to the pre-operative images (*p* < 0.05).

**Figure 8:**
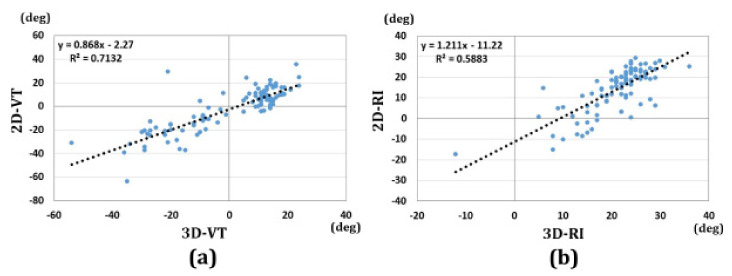
Results of the correlations between 2D and 3D measurements. (**a**) Results of the correlations between 2D-VT and 3D-VT. (**b**) Results of the correlations between 2D-RI and 3D-RI.

The correlations between 3D-VT and 2D-VT, and between 3D-RI and 2D-RI, are shown in Figure 8. The correlation coefficients were 0.84 and 0.77 for the volar tilt and radial inclination, respectively. There were significant correlations between 2D and 3D measurements.

## 4. Discussion

In this study, we attempted to develop a computer-aided three-dimensional analysis method to express displacements and reductions of distal radius fractures. Some previous studies evaluated distal radius fracture patterns with computed tomography [7,8,17,18,19]. In those studies, it was suggested that there were different patterns of location and directions of fractures according to the volar/dorsal displacements of the distal radius. Namely, dorsal displacement fractures are more common in the dorsoulnar segment and less common in the dorsoradial segment. On the other hand, volar displacement fractures always involve the scaphoid facet [19]. In another study, it was suggested that an articular fracture of distal radius was more likely to occur between the ligament attachments [20]. There was also another attempt to assess articular comminution by the articular surface area of the distal radius or heat mapping of the fracture lines [21]. More recently, the concept of key fragments has been described [22]. It has been suggested that the degree of gap and step off of the radius joint surface differs depending on the fracture patterns of the dorsal ulnar corner and dorsal wall relative to the ulnar-palmar fragment. The finding that the ulnar side reference points moved less than the reference points of the radial styloid process may support this concept. Through these 3D evaluations of distal radius fractures, the fracture patterns could be analyzed stereoscopically on the basis of anatomical landmarks. Although these studies attempted to describe three-dimensional characteristics of distal radius fractures, due to the lack of standardized expressions based on 3D coordinates, the degree of dislocation and the accuracy of reduction could not be compared on the same standard. For the appropriate treatment or surgical planning of distal radius fractures, developing an analysis method of radius shape based on 3D coordinates is considered urgent.

In our study, on the basis of the long axis of the intact part of the distal radius, we tried to develop a method to express the displacement of distal radius fractures three-dimensionally. Three-dimensional characterizations of the distal radius fractures were achieved by measuring three reference points that were relatively clear on the anatomical landmarks. As a result, it was found that the reliability of the three reference points was very high. We also evaluated barycentric coordinates of three reference points to express the direction and degree of displacement. With these landmarks and parameters, it is thought that the dislocation and instability of fractures can be easily recognized. Although the centroid of the three reference points is not the centroid for force transmission, several biomechanical studies suggest that the centroid of the force transmission in the distal radius articular surface is located on the palmar side of the volar lunate facet [23,24,25,26,27]. This is relatively close to the position of the centroid of the three reference points shown in the postoperative images in this study. Thus, in reference to the radius long axis, the centroid of the three reference points can be a parameter to confirm if the center of the force transmission in the wrist has been reconstructed by the reductions. In addition, the 3D parameters of VT and RI were well correlated with the 2D parameters. Although the measurement methods were different in the 2D and 3D images, there were excellent correlations. With feedback of 3D measurement results to 2D measurements, it may be possible to facilitate 2D evaluations. In addition to these parameters, the plane area of three reference points can be evaluated. Since there was a significantly smaller plane area on the post-operative images compared to the pre-operative images, this can be a parameter for the reconstructions of the articular surface gap. The method described in our study is a way to standardize and express the displacements and reductions of distal radius fractures three-dimensionally. This method and the reference points may be helpful in understanding the three-dimensional direction and extent of displacements in the distal radius fractures. In case of severely displaced fractures, it is sometimes difficult to estimate the distance and the directions of reduction preoperatively. These quantifications are not available in the conventional two-dimensional evaluations. Since we could understand how much and which direction to move the reference points, these measurements may be helpful in simplifying and automating fracture reductions.

There were several limitations in this study. First, a computer software is required to perform the analysis described in this study. If this method is accepted generally, it may be possible to develop a program to automatically choose the reference points and measure the directions of displacements in the general image viewer. Second, the origin of the coordinate was set on the intersection of the joint surface and the radius long axis in the postoperative images. To know the reduction shape preoperatively, the unaffected side of the image may require to be used when setting the origin. The most important comparison values for a sufficient restoration of a fractured joint are the values of non-injured joints. In this study, CT scans of the uninjured wrist were not performed, due to the problems of radiation exposure and the optimization of medical expenses. This may need to be solved in future studies.

## 5. Conclusions

We developed a three-dimensional analysis method for the determination of displacements and reductions of distal radius fractures. This method and the associated reference points may be helpful in understanding the three-dimensional direction and extent of displacements in distal radius fractures. It is also helpful to estimate the accuracy of the reductions in the treatments. This computer-aided method is one approach to clarify in which direction and how much each reference point needs to be reduced with respect to the radius long axis.

## Data Availability

The datasets used and/or analyzed during the current study are available from the corresponding author on reasonable request.
